# Maternal Mental Health and Child Dietary Diversity in Rural Kenya: Findings From a Pooled Analysis of 2 Baseline Studies

**DOI:** 10.1016/j.cdnut.2025.107497

**Published:** 2025-06-24

**Authors:** Md Abul Kalam, Juliet K. McCann, Zarmeen Shakil, Aishat Gambari, Michael Ochieng, Joshua Jeong

**Affiliations:** 1 Global Health and Development Program, Laney Graduate School, Emory University, Atlanta, GA, United States; 2 Hubert Department of Global Health, Rollins School of Public Health, Emory University, Atlanta, GA, United States; 3 Nutrition and Health Sciences Program, Laney Graduate School, Emory University, Atlanta, GA, United States; 4 B&M Consult, Nairobi, Kenya

**Keywords:** maternal mental health, parenting stress, depression, child nutrition, child diet, LMICs, Kenya

## Abstract

**Background:**

Most studies linking maternal mental health and child outcomes are from high-income countries and relatively few studies have explored how maternal mental health influences child nutrition in impoverished and rural settings across low-income countries.

**Objectives:**

This study aimed to assess the association between maternal mental health and child dietary diversity (CDD) in rural western Kenya.

**Methods:**

The analysis pooled baseline data from 2 RCTs of nurturing care interventions in rural western Kenya (clinical trial registrations are clinicaltrials.gov: NCT05796934 and clinicaltrials.gov NCT06165315, respectively). The 2 primary exposure variables were binary indicators for elevated maternal parenting stress and risk of maternal depression, which were self-reported using Parenting Stress Index—Short Form and Center of Epidemiologic Studies Depression Scale, respectively. The outcome was CDD, assessed using the World Health Organization measure for dietary diversity, which was based on maternal reports of the number of food groups consumed by the child in the past 24 h. Multivariable linear regression analyses were used to examine the association between maternal parenting stress, maternal depression and CDD.

**Results:**

The analytic sample was 690 mother–child dyads. The mean maternal age was ∼28 y (range: 17–49 y), whereas the mean child age was ∼14 mo (range: 6–25 mo). The mean dietary diversity score was 4.40 of 8 food groups. Approximately 20.14% of mothers had elevated parenting stress, whereas 41% were at risk of depression. The adjusted models showed that elevated maternal parenting stress was associated with lower CDD (β: −0.39, 95% CI: −0.80, 0.02; *P* = 0.059), whereas the association was not statistically significant between risk of maternal depression and CDD (β: 0.14, 95% CI: −0.14, 0.43; *P* = 0.323). Multiple sociodemographic factors were significantly associated with CDD. Children of mothers with higher social support, older children (range: 6–25 mo), and female children had higher dietary diversity. Meanwhile children from high food-insecure households had lower dietary diversity.

**Conclusions:**

These findings underscore the importance of integrating psychosocial components within child nutrition programs to address not only maternal mental health but also support the broader caregiving environment for families in low-and-middle-income settings like Kenya. Strengthening caregivers’ social support networks as part of these interventions may also have potential for promoting maternal mental health and children’s nutritional well-being.

## Introduction

Maternal mental health encompasses multiple dimensions, including maternal depression and stress [1]. Poor mental health has been found to be particularly prevalent among mothers with young children due to not only to biological factors related to pregnancy and childbirth but also to psychosocial stressors and females’ roles as primary caregivers [2,3]. A recent meta-analysis conducted across 46 countries estimated the global pooled prevalence of maternal depression at ∼20% [4] with a higher burden at 24.7% in lower-and-middle-income countries (LMICs) than that in high-income countries (HICs) [5,6]. In sub-Saharan Africa specifically, the prevalence of postpartum depression was estimated at 18.6% [7]. Parenting stress, another domain of maternal mental health, has also been shown to contribute to maternal depression and other psychological problems among females, as well as adverse child outcomes [8].

Most of the evidence on the adverse effects of maternal mental health problems on young children is based on research conducted in HICs and primarily from the fields of child psychology and psychiatry [[9], [10], [11], [12]]. Yet, poor maternal mental health is a major global health concern that can compromise various outcomes for children living in LMICs. For example, child malnutrition along with poor child development is a significant public health challenge in LMICs, where 91% of total stunted children under 5 y of age are living and nearly 2 of 3 children are not meeting their minimum dietary diversity [13,14]. Several longitudinal studies and meta-analyses have documented that maternal depression increased risk of child underweight and stunting, particularly in LMICs [[15], [16], [17]]. Other studies have also shown links between maternal depression and infant and young child feeding practices like breastfeeding, responsive feeding, and other complementary feeding practices [[18], [19], [20], [21]]. For example, a study in Ethiopia found that higher postnatal depression was associated with lower scores on the infant feeding practices [18]. Similarly, a study in rural Malaysia found that mothers with depression were more likely to stop exclusive breastfeeding earlier than mothers without depression [21]. However, it is also worth noting that the evidence is mixed as some studies have also discovered null associations between maternal depression and various child nutrition outcomes, including stunting, breastfeeding, and complementary feeding indicators [[22], [23], [24]]. For example, a study in northern Ghana showed that maternal depression did not affect child minimum dietary diversity, minimum meal frequency, and minimum acceptable diet [22]. These inconsistencies may reflect differences due to the study design or contextual factors across settings.

In addition to the direct influence of maternal mental health, household, partner, and social or community characteristics also influence child nutritional outcomes. Socioeconomic status and food insecurity have been identified as significant risk factors for maternal depression and poor child dietary diversity (CDD) [[25], [26], [27]], whereas social support—especially spousal support—can buffer the negative consequences of maternal mental health problems among the mothers of young children [[28], [29], [30]]. Active involvement of fathers in child feeding has been shown to enhance children’s feeding practices—such as exclusive breastfeeding, extended breastfeeding duration, and improved dietary diversity—highlighting the essential role fathers play within the family system and their crucial support in bolstering maternal efforts [[31], [32], [33], [34]]. Yet, few studies have considered these factors with respect to the relationship between maternal depression or parenting stress and child feeding practices.

In Kenya, child undernutrition remains a significant public health concern among children under 5 y. According to the 2022 Kenya Demographic and Health Survey, 18% of children under 5 y were stunted, with a higher prevalence in rural areas than that in urban areas (20% vs 12%) [35]. In terms of CDD, 71% of children aged 6–23 mo were fed the minimum number of times deemed appropriate for their age, whereas only 37% were reported to have an adequately diverse diet and 31% met the minimum acceptable diet [35].

Similar to other LMICs, there is a lack of national data on maternal depression and parenting stress in Kenya. However, cross-sectional studies have reported that the prevalence of maternal depression ranges between 13.0% and 27.1% among Kenyan mothers [[36], [37], [38]]. Compared with infants of mothers without depression, infants of mothers with depression tend to experience poorer physical and neurocognitive development along with a higher likelihood of illnesses [39]. Yet, maternal depression and parenting stress remain underrecognized and understudied especially with regards to complementary feeding in Kenya, particularly in rural areas. Additionally, there is also a knowledge gap on how the broader caregiver environment such as fathers’ involvement and maternal social support may contribute to CDD in Kenya. Therefore, this study aimed to explore the relationship between 2 exposures: *1*) risk of maternal depression and *2*) elevated maternal parenting stress and child feeding practices, characterized by CDD in rural western Kenya. Understanding these dynamics is crucial for devising effective and equitable interventions to promote maternal mental health and children’s nutritional well-being in LMIC settings like Kenya.

## Methods

### Study design and settings

This cross-sectional study uses secondary data analysis, combining baseline data from 2 ongoing cluster RCTs on parenting programs in rural western Kenya; the Moments that Matter (MTM) program (clinicaltrials.gov: NCT05796934) and the ChildFund trial (clinicaltrials.gov #NCT06165315). First, the MTM trial is a community-based, multicomponent parenting program delivered to primary caregivers of children under 3 y of age in Nyamia and Vihiga counties. This project is being implemented by Anglican Development Services (ADS)-Nyanza and Western. Four project sites were randomly selected within each subcounty and divided into intervention or waitlist control groups. In total, 46 villages were included in the main MTM study with stratification by county. Within each village, 13 households were randomly selected based on the following inclusion criteria: primary caregivers of a child aged 0–18 mon, residing within the selected village, and providing informed consent for themselves and their child(ren). Considering these criteria, the main trial included 595 primary caregiver–child dyads (3 households were excluded due to nonconsent to participate in the trial).

Second, the ChildFund trial is another community-based parenting program that is being delivered through existing community group network to improve early childhood development in HomaBay and Busia counties [40]. Nyamira subcounty in HomaBay county and Bunyala subcounty in Busia County were selected by the implementing partners as the project sites. Sixty-four villages were included in the study with stratification by county. In each village, 10 primary caregivers (with an additional 2 caregivers in 2 villages) with a child aged 0–24 mo were randomly selected, resulting in 642 caregiver–child dyads enrolled in the main trial, which were divided into intervention or waitlist control groups.

For both trials, the selection of counties and subcounties was guided by government priorities, particularly in areas with poor maternal and child health and nutrition indicators. Based on these criteria, the final selection of subcounties, project sites, and villages was made collaboratively by implementing partners, government stakeholders, and the Emory University research team.

### Participants and procedure

**FIGURE 1:**
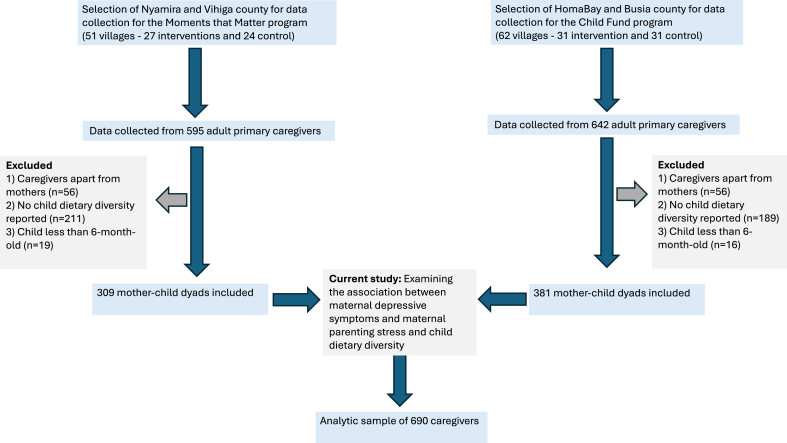
Participant diagram depicting the flow of participants and analytic sample from the study population.

The sample was restricted to mothers who had a child between 6 and 25 mo of age. From the initial pool, data were collected from 595 adult primary caregivers in Nyamira and Vihiga counties for the MTM trial and 642 in Homabay and Busia counties for the ChildFund trial. For inclusion in this analysis, only mother–child dyads were considered, specifically where the caregiver was the mother, the child was ≥6 mo old, and CDD information was available. In Nyamira and Vihiga, 56 caregivers who were not mothers, 212 dyads with no CDD data, and 19 dyads with children younger than 6 mo were excluded. Similarly, in HomaBay and Busia, 56 nonmother caregivers, 189 dyads lacking CDD data, and 16 dyads with children under 6 mo were excluded. After applying these criteria, 309 mother–child dyads from Nyamira/Vihiga and 381 from HomaBay/Busia were included, resulting in an analytic sample of 690 mother–child dyads for this analysis. The inclusion has been presented in Figure 1.

### Data collection

Data were collected for the MTM baseline evaluation by a team of Kenya-based research assistants from February to March, 2023. For the ChildFund evaluation, data were collected from October to November, 2023 by another team of Kenya-based research assistants. For both trials, data collection was overseen by 2 Kenyan supervisors. Research assistants held bachelor’s degrees and were selected based on having previous relevant field-based research experience and being originally from the counties within which they would be collecting data. Prior to data collection, all research assistants received 7-d (MTM) or 9-d (ChildFund) training, which included both classroom instruction and supervised field-based practice. Both surveys’ durations were ∼1–2 h and were administered verbally in Kiswahili in a private location within participants’ households. Data were collected via Android mobile devices using the Open Data Kit application. In both trials, the baseline was conducted using the same tools to measure the outcome, exposures and covariates as outlined further.

### Measures

**FIGURE 2:**
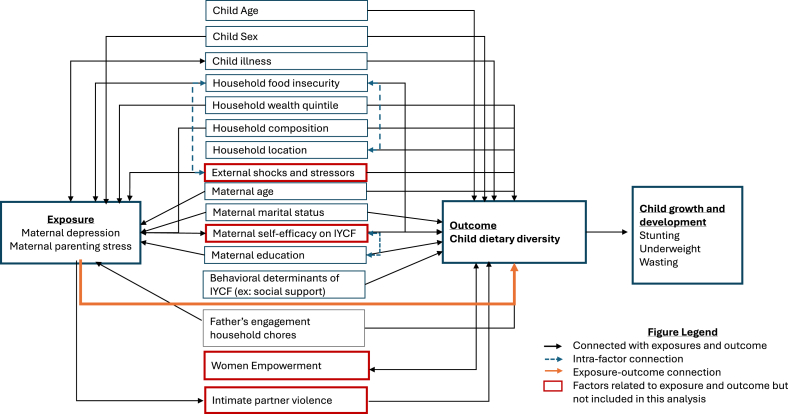
Conceptual framework depicting the relationship between maternal depression, maternal parenting stress, child dietary diversity, and a variety of other sociodemographic and household factors.

The 2 primary exposures examined in this study were maternal depression and maternal parenting stress, as illustrated in Figure 2, based on a literature review. Maternal depression was self-reported using the Center of Epidemiologic Studies Depression Scale, 10-item questionnaire (CESD-10) [29]. Respondents were asked to indicate their frequency of experiencing 10 common symptoms of depression (the items included, “I feel too down to do anything,” “I felt lonely,” “I felt happy,” “I did not sleep as well as I usually sleep,” “I felt worried or afraid without any special reason,” “I felt hopeful that my life will improve,” “I felt that everything I did was an effort, I felt too tired even to do small chores ,” “I felt down and unhappy,” “I could not pay attention to what I was doing because I kept thinking about my sadness and worries,” and “I was bothered by things that usually do not bother me”) in the past week on a 4-point Likert scale. Details of this approach have been described elsewhere [29]. Total scores thus ranged from 0 to 30, with higher scores indicating greater severity of depression. The CESD-10 had a good internal consistency (Cronbach α: 0.73). A binary indicator risk of maternal depression was defined using a cutoff of ≤10. The CESD-10 has been previously validated in Kenya and found to have strong diagnostic performance in terms of both its sensitivity and specificity in identifying risk of depression among postpartum females [41].

Maternal stress was defined as the extent to which mothers feel competent, restricted, conflicted, supported, and/or depressed in their role as a parent. This was measured using the Parenting Stress Index-Short Form [42]. The Parental Stress subscale included 12 items (“Do you feel like you can keep things under control well?”, “Do you feel like you often make greater sacrifices in your life than what’s possible in reality in order to satisfy the needs of your child?”, “Do you feel trapped by your parental responsibilities?”, “After becoming a caregiver of your child, do you feel more unhappy now while doing things that you used to enjoy?”, “After becoming a caregiver of your child, do you feel like you can still do what you enjoy?”, “After becoming a caregiver of your child, do you feel like you can still do new and different things?” “Are there many things bothering you in your life?”, “Has becoming a caregiver to your child caused more problems than you expected in the relationship between you and your spouse/partner?”, “Do you feel lonely and like you have no friends?”, “Do you believe you won’t have a good time when you go out?”, “Do you have less interest in socializing with people than before?”, and “Do you enjoy things less than before?”), with each rated on a 5-point Likert scale ranging from 1 (strongly agree) to 5 (strongly disagree). Negatively worded items were reverse coded, and a total score was calculated by summing the responses to all 12 items. The Parenting Stress Index-Short Form scale in this study showed a reliable and good internal consistency (Cronbach α: 0.78). Elevated maternal stress was dichotomized into low and high stress, where a score of 0–43 represented low stress and a score ≥44 represented high stress. The cutoff was defined based on the 81st percentile of all scores per scoring guidelines of the original tool [43].

### Outcome

CDD was assessed using the WHO Infant and Young Child Feeding indicator for child minimum dietary diversity, evaluating the number of distinct food groups consumed by the child in the previous 24 h. [44]. Following WHO guidelines, we collected information from the mothers using the 24-h recall questionnaire and classified foods into 8 groups: *1*) breast milk, *2*) grains, roots, and tubers, *3*) legumes and nuts, *4*) dairy products (milk, yogurt, and cheese), *5*) flesh foods (meat, fish, poultry, and organ meats), *6*) eggs, *7*) vitamin A–rich fruits and vegetables, and *8*) other fruits and vegetables. A continuous dietary diversity score (DDS) ranging from 0 to 8 was calculated by summing the number of food groups consumed. It indicated whether a child aged 6 mo or older received minimum dietary diversity in the past 24 h and was validated as a proxy measure of nutrient adequacy by WHO.

### Covariates

This study included a set of sociodemographic and psychosocial covariates including fathers’ involvement in household chores, maternal social support, household food security status, child age, child sex, breastfeeding status, presence of siblings under 5, maternal age, maternal marital status, maternal education, household wealth quintile, and geographic locations.

#### Fathers’ involvement

The survey tool to measure fathers’ involvement in household chores was adapted from previous measures of father involvement in child nutrition and caregiving in similar contexts in East Africa [[45], [46], [47]]. This was reported by mothers and comprised various activities pertaining to fathers’ engagement in household chores (including prepared or helped partner prepare food, collected firewood, washed clothes, bathed or helped partner to bathe child, washed dishes, prepared tea for household, and swept home compound or cleaned house) in the past 2 wk. The psychometric property of this scale showed good internal consistency reliability and evidence of multiple forms of validity in these settings [48]. We calculated summative score of fathers’ involvement score by the number of household activities (range: 0–7) they performed, with higher scores indicating greater levels of involvement. The items of this measurement tool and other details are described elsewhere [29].

#### Maternal social support

Maternal social support was assessed using the 12-item Multidimensional Scale of Perceived Social Support [49]. The scale included 3 subscales with 4 items each, capturing support from *1*) partner or special person (items included “Is there is a special person who is around when you are in need?”, “Is there is a special person with whom you can share your joys and sorrows?”, “Do you have a special person who is a real source of comfort to you?”, and “Is there a special person in your life who cares about your feelings?”); and *2*) family (items included “Does your family really try to help you when you need help or support?”, “Can you talk about your problems with your family?”, “Is your family willing to help you to make decisions?”, and “Do you get the emotional help and support you need from your family?”) and friends (items included “Can you count on your friends when things go wrong?”, “Do you have friends with whom you can share my joys and sorrows”, “Can you talk about your problems with your friends?”, and “Can you count on your friends when things go wrong?”). Responses for each item were scored on a 5-point Likert scale with 1 representing strongly disagree and 5 strongly agree. A total perceived maternal social support score was calculated by averaging responses across all 12 items, with higher scores indicating greater perceived support. The maternal social support scale demonstrated good internal consistency (Cronbach α: 0.82).

The study also included 6 covariates based on literature, theoretical justification, and tests of correlation between variables of interest. These covariates included household food insecurity, child age, child sex, siblings under 5 y, maternal age, maternal marital status, maternal education, wealth quintile, and geographic locations of residency (county). Household food security was assessed using 2 caregiver-reported items regarding the frequency of going to sleep hungry and going a whole day without eating due to lack of food. These items were adapted from food insecurity experience scale that was developed by FAO [50]. Responses were scored on a 4-point scale [1 = never, 2 = rarely (1–2 times), 3 = sometimes (3–10 times, and 4 = often (>10 times)], and the total score, summing both items, reflected the level of food insecurity, with higher scores indicating greater food insecurity. Child age was a continuous variable (in months) and child sex was binary (male/female); maternal age was categorized into groups (18–24, 25–34, 35–44, and ≥45 y). Maternal marital status was recoded as never married, married, cohabitating, and other. Maternal education was categorized as no education, some primary school (those who did not complete primary education level, i.e., ≤seventh grade), primary school completed, and secondary school completed. Wealth quintile was assessed using a categorical asset quintile, ranging from the lowest (first quintile) to the highest (fifth quintile). The wealth quintile was derived using a composite index incorporating housing characteristics (including roof and floor type) and ownership of a range of small and medium assets—such as electricity, solar panels, radios, televisions, mobile and landline phones, refrigerators, various furniture items, kitchenware, watches, and modes of transportation (bicycles, cars, and motorbikes). Principal components analysis was applied to these variables to generate a continuous wealth score, which was then divided into quintiles to categorize households into 5 relative wealth groups.

### Statistical analyses

A descriptive analysis of key study variables was conducted, which included our 2 exposures, outcome, demographic variables, and the covariates. These included maternal depression and parenting stress, CDD, maternal social support and father involvement, as well as maternal age, marital status, education, along with child-related factors such as sex, age, breastfeeding status, and siblings under 5 y of age. We also examined distributions in household-level factors such as asset quintile, food security, county, and family composition to provide a holistic understanding of our study context. We applied χ^2^ and *t* tests to compare the sample characteristics by trials (Supplemental Table 1).

We conducted 2 adjusted linear regression analyses for 2 exposures, controlling for sociodemographic variables informed by insights from previous literature and adjusting for county. The demographic characteristics of respondents in the ChildFund trial differed significantly from those in the MTM trial (see Supplemental Table 1 for a comparison of sociodemographic characteristics by trial). Both adjusted models accounted for these systematic differences and the dummy variables for each county of residence. The SEs were clustered at the (cluster variable) level in both models.

In the first model, we estimated the association between maternal parenting stress and CDD using a linear regression model adjusted for maternal social support, fathers’ involvement in household chores, child age and sex, presence of siblings under 5 y, marital status, caregiver age and education, household asset quintile, and food insecurity using the following equation:

(1) $${ChildDDS}_{i}=\beta_{0}+\beta_{1}.{PSI}_{i}+\beta_{2}+\ldots +\beta_{12}+\epsilon_{i}\;\text{.}$$

where ${ChildDDS}_{i}$ is the DDS for child i, ${PSI}_{i}$ is the maternal parenting stress for individual mother of child i, $\beta_{2}+\ldots +\beta_{12}$ are the covariates, and $\epsilon_{i}$ is the error term, clustered at the study cluster level.

Similarly, we estimated the association between maternal depression and CDD using another linear regression model adjusted for maternal social support, fathers’ involvement in household chores, child age and sex, presence of siblings under 5 y, marital status, caregiver age and education, household asset quintile, food insecurity, and county of residence, using the following equation:

(2) $${ChildDDS}_{i}=\beta_{0}+\beta_{1}.{CESD}_{i}+\beta_{2}+\ldots +\beta_{12}+\epsilon_{i}$$

where ${ChildDDS}_{i}$ is the DDS for child i, ${CESD}_{i}$ is the maternal depression for individual mother of child i, $\beta_{2}+\ldots +\beta_{12}$ are the covariates, and $\epsilon_{i}$ is the error term, clustered at the study cluster level.

Statistical significance of associations was assessed using *P* values, with a threshold of *P* < 0.05. All analyses were conducted in Stata 17.0 (StataCorp).

## Results

### Sample characteristics

The analytic sample size consisted of 690 mother–child pairs. Table 1 shows that the mean age of children was 13.7 mo (SD: 5.02 mo; range: 6–25 mo), a little more than half of the children were female (51.2%), ∼76% of children continued breastfeeding, and ∼35% children had a sibling under 5 y of age with the highest number of siblings being 2 (*n* = 18). Approximately 32% of mothers were <25 y of age, and 43.48% were married, whereas 36% were cohabitating. Two in 5 mothers (41%) completed the primary level of education. The sample was not equally distributed across the 4 counties (range: 21.6%–28.6%). Similarly, the household wealth status was also not distributed equally (19.3%–20.6%). Slightly under half (46.8%) of the households reported some level of food insecurity. The overall perceived maternal social support score was 3.45 of 5. Mothers also reported that ∼53% of fathers engaged in ≥1 household chore.

**TABLE 1:** Description of demographic characteristics, exposures, and outcome in the analytic sample.

Variable	Value^1^
Demographic characteristics	
Child age (mo)	13.68 ± 5.02; 6–25
Child sex	
Female	353 (51.16)
Male	337 (48.84)
Any breastfeeding	
Yes	525 (76.09)
No	165 (23.91)
Siblings under 5 y	
No sibling	446 (64.64)
1 sibling	226 (32.75)
2 siblings	18 (2.61)
Maternal age (y)	27.89 ± 5.92; 17–49
18–24	220 (31.88)
25–34	356 (51.59)
35–44	108 (15.65)
45+	6 (0.87)
Maternal marital status	
Never married	91 (13.19)
Married	300 (43.48)
Cohabitating	251 (36.38)
Other	48 (6.96)
Maternal education	
No education	5 (0.72)
Some primary school^2^	173 (25.07)
Primary school completed	283 (41.01)
Secondary school completed	229 (33.19)
Wealth quintile (1–5)	
Lowest	133 (19.28)
Second	140 (20.29)
Middle	138 (20.00)
Fourth	142 (20.58)
Highest	137 (19.86)
County	
HomaBay	184 (26.67)
Busia	197 (28.55)
Nyamira	160 (23.19)
Vihiga	149 (21.59)
Food insecurity	
None	367 (53.19)
Low	110 (15.94)
Moderate	106 (15.36)
High	107 (15.51)
Fathers’ involvement in household chores	1.62 ± 2.09; 0–7
Maternal social support	3.46 ± 0.64; 1–4.92
Outcome variable	
Child dietary diversity (0–8)	4.40 ± 1.63; 1–8
Child minimum dietary diversity	
5 and more	377 (54.64)
Less than 5	313 (45.36)
Exposures	
Maternal stress	
Low stress (PSI <43)	551 (79.86)
High stress (PSI ≥44)	139 (20.14)
Depression	
Low symptoms (CESD < 10)	410 (59.42)
High symptoms (CESD ≥ 10)	280 (40.58)

Abbreviations: CESD, Center for Epidemiologic Studies Depression Scale; PSI, Parenting Stress Index.

1 Values are Mean ± SD and range for the continuous variables and % for categorical variables.

2 Some primary was coded when respondents did not complete primary level of education (i.e., seventh grade).

The mean DDS was 4.40 of 8, and ∼55% of the children consumed 5 or more food groups. Approximately 20.14% of mothers had elevated parenting stress, whereas 41% of mothers were at risk of depression.

### Associations between maternal mental health and CDD

Table 2 shows the results for the relationship between elevated maternal parenting stress and CDD. In the unadjusted model, maternal parenting stress was inversely associated with CDD (β: −0.54; 95% CI: −0.82, −0.27; *P* < 0.001). After adjusting the covariates, maternal parenting stress remained associated with lower dietary diversity although the magnitude of the association was attenuated (β: −0.39; 95% CI: −0.80, 0.02; *P* = 0.059).

**TABLE 2:** Association between parenting stress and CDD.

Variables	Unadjusted results for maternal stress and CDD			Adjusted results for maternal stress and CDD		
	β (95% CI)	SE	*P*	β (95% CI)	SE	*P*
Maternal stress (reference: low stress)	−0.54 (−0.82, −0.27)	0.14	<0.001	−0.39 (−0.80, 0.02)	0.20	0.059
Social support	—	—	—	0.16 (−0.08, 0.40)	0.12	0.191
Father’s involvement in household chores	—	—	—	0.04 (−0.03, 0.12)	0.04	0.282
Child age (mo)	—	—	—	0.03 (0.002, 0.06)	0.02	0.034
Child sex (reference: female)	—	—	—	−0.38 (−0.62, −0.14)	0.13	0.002
Siblings under 5 y (reference: no sibling)						
1 sibling	—	—	—	0.07 (−0.18, 0.32)	0.13	0.561
2 siblings	—	—	—	0.35 (−0.40, 1.10)	0.38	0.357
Marital status (reference: never married)						
Married	—	—	—	−0.18 (−0.66, 0.30)	0.24	0.451
Cohabitating	—	—	—	0.53 (0.04, 1.02)	0.25	0.034
Other	—	—	—	0.21 (−0.60, 1.01)	0.40	0.610
Maternal age (reference: 18–24 y)						
25–34	—	—	—	0.03 (−0.26, 0.32)	0.15	0.849
35–44	—	—	—	0.31 (−0.10, 0.73)	0.21	0.138
45 and above	—	—	—	0.50 (−0.54, 1.54)	0.52	0.338
Maternal education (reference: no education)						
Some primary school^1^	—	—	—	0.83 (0.30, 1.36)	0.27	0.003
Completed primary education	—	—	—	1.01 (0.51, 1.51)	0.25	<0.001
Completed secondary education	—	—	—	0.93 (0.35, 1.51)	0.29	0.002
Asset quintile (reference: lowest quintile)						
Second quintile	—	—	—	0.35 (−0.03, 0.74)	0.19	0.074
Middle quintile	—	—	—	0.63 (0.28, 0.98)	0.18	0.001
Fourth quintile	—	—	—	0.70 (0.26, 1.15)	0.22	0.002
Highest quintile	—	—	—	0.87 (0.43, 1.32)	0.22	<0.001
Food insecurity (reference: none)						
Low	—	—	—	−0.13 (−0.41, 0.15)	0.14	0.352
Moderate	—	—	—	−0.35 (−0.74, 0.04)	0.19	0.074
High	—	—	—	−0.56 (−1.00, −0.12)	0.22	0.014
County (reference: HomaBay)						
Busia	—	—	—	0.13 (−0.31, 0.57)	0.22	0.553
Nyamira	—	—	—	−0.26 (−0.81, 0.29)	0.26	0.347
Vihiga	—	—	—	−0.45 (−0.92, 0.02)	0.24	0.063

Abbreviations: CDD, child dietary diversity.

1 Some primary was coded when respondents did not complete primary level of education (i.e., seventh grade).

Table 3 shows the results for the relationship between risk of maternal depression and CDD. In the unadjusted model, the analysis revealed a negative but not statistically significant association between maternal depression and CDD (β: −0.19, 95% CI: −0.45, 0.05; *P* = 0.119), which attenuated to a small positive association (β: 0.14, 95% CI: −0.14, 0.43; *P* = 0.323) in the adjusted model, albeit a null association.

**TABLE 3:** Association between maternal depression and CDD.

Variables	Unadjusted results for maternal depression and CDD			Adjusted results for maternal depression and CDD		
	β (95% CI)	SE	*P*	β (95% CI)	SE	*P*
Maternal depression	−0.19 (−0.45, 0.05)	0.13	0.119	0.14 (−0.14, 0.43)	0.14	0.323
Social support	—	—	—	0.23 (−0.01, 0.47)	0.12	0.042
Father’s involvement in household chores	—	—	—	0.04 (−0.03, 0.12)	0.04	0.239
Child age (mo)	—	—	—	0.03 (0.002, 0.06)	0.02	0.037
Child sex (reference: female)	—	—	—	−0.38 (−0.61, −0.15)	0.12	0.002
Siblings under 5 y (reference: no sibling)						
1 sibling	—	—	—	0.04 (−0.21, 0.29)	0.13	0.761
2 siblings	—	—	—	0.28 (−0.49, 1.04)	0.38	0.472
Marital status (reference: never married)						
Married	—	—	—	−0.17 (−0.64, 0.30)	0.24	0.476
Cohabitating	—	—	—	0.53 (0.03, 1.03)	0.25	0.039
Other	—	—	—	0.09 (−0.72, 0.90)	0.41	0.825
Maternal age (reference: 18–24 y)						
25–34	—	—	—	0.03 (−0.26, 0.31)	0.14	0.859
35–44	—	—	—	0.31 (−0.10, 0.71)	0.20	0.133
45 and above	—	—	—	0.46 (−0.66, 1.59)	0.56	0.414
Maternal education (reference: no education)						
Some primary school^1^	—	—	—	0.65 (0.15, 1.16)	0.25	0.012
Completed primary education	—	—	—	0.88 (0.39, 1.36)	0.24	0.001
Completed secondary education	—	—	—	0.80 (0.23, 1.37)	0.29	0.007
Asset quintile (reference: lowest quintile)						
Second quintile	—	—	—	0.39 (0.01, 0.76)	0.19	0.044
Middle quintile	—	—	—	0.68 (0.33, 1.02)	0.17	<0.000
Fourth quintile	—	—	—	0.75 (0.32, 1.19)	0.22	0.001
Highest quintile	—	—	—	0.91 (0.46, 1.35)	0.22	<0.000
Food insecurity (reference: none)						
Low	—	—	—	−0.12 (−0.40, 0.16)	0.14	0.405
Moderate	—	—	—	−0.39 (−0.77, 0.001)	0.19	0.050
High	—	—	—	−0.60 (−1.06, −0.14)	0.23	0.011
County (reference: HomaBay)						
Busia	—	—	—	0.22 (−0.21, 0.64)	0.21	0.311
Nyamira	—	—	—	−0.11 (−0.63, 0.42)	0.26	0.685
Vihiga	—	—	—	−0.28 (−0.70, 0.14)	0.21	0.191

CDD, child dietary diversity.

1 Some primary was coded when respondents did not complete primary level of education (i.e., seventh grade).

### Other sociodemographic correlates with CDD

In the adjusted analyses, we found that several factors were significantly correlated with CDD above and beyond maternal mental health. The common factors in both models included child age, child sex, maternal education level, wealth quintiles, and household food insecurity status. Specifically, in both models, the older children had higher DDSs (in the adjusted maternal parenting stress model—β: 0.03; 95% CI: 0.002, 0.06; *P* = 0.034; in the adjusted depression model—β: 0.03; 95% CI: 0.002, 0.06; *P* = 0.037) than the younger children (age range: 6–25 mo). Male children had lower DDSs than the female children (in the adjusted model with maternal parenting stress—β:−0.38; 95% CI: −0.62, −0.14; *P* = 0.002; in the adjusted model with maternal depression model—β: −0.38; 95% CI: −0.61, −0.15; *P* = 0.002). Compared with children of mothers who had never married, those whose mothers were cohabiting with a partner had higher dietary diversity (in the adjusted maternal parenting stress model—β: 0.53; 95% CI: 0.04, 1.02; *P* = 0.034; in the adjusted depression model—β: 0.53; 95% CI: 0.03, 1.03; *P* = 0.039).

Higher maternal education was significantly associated with greater dietary diversity than no formal education. In the maternal parenting stress model, having some primary education (β: 0.83; 95% CI: 0.30, 1.36; *P* = 0.003), completed primary education (β: 1.01; 95% CI: 0.51, 1.51; *P* < 0.001), and completed secondary education (β: 0.93; 95% CI: 0.35, 1.51; *P* = 0.002) were positively associated with higher dietary diversity. Similar patterns were observed in the depression model, with some primary education (β: 0.65; 95% CI: 0.15, 1.16; *P* = 0.012), completed primary education (β: 0.88; 95% CI: 0.39, 1.36; *P* = 0.001), and completed secondary education (β: 0.80; 95% CI: 0.23, 1.37; *P* = 0.007) showing significant associations with higher CDD.

Household wealth was also positively associated with CDD. Compared with children in the lowest wealth quintile, those in higher quintiles had significantly higher DDSs. In the maternal parenting stress model, children in the middle wealth quintile had β of 0.63 (95% CI: 0.28, 0.098; *P* = 0.001), fourth quintile had β of 0.70 (95% CI: 0.26, 1.15; *P* = 0.003), and those in the highest quintile had a β of 0.87 (95% CI: 0.43, 1.32; *P* < 0.001). Similarly, in the maternal depression model, children in the fourth quintile had a β of 0.75 (95% CI: 0.32, 1.19; *P* = 0.001), and those in the highest quintile had a β of 0.91 (95% CI: 0.46, 1.35; *P* < 0.001).

Food insecurity was also a significant risk factor of lower CDD. Compared with children who were not from food-insecure households, those from high food insecure (in the stress model—β: −0.56; 95% CI: −1.00, −0.12; *P* = 0.014; in the depression model—β: −0.60; 95% CI: −1.06, −0.14; *P* = 0.011) and those from moderate food insecure (in the depression model—β: −0.39; 95% CI: −0.77, 0.001; *P* = 0.050) had lower dietary diversity.

Finally, the adjusted model on depression revealed that children whose mothers reported greater social support had higher dietary diversity (β: 0.23; 95% CI: −0.01, 0.47; *P* = 0.042) than those who reported less social support.

## Discussion

In this study, we examined the associations between maternal parenting stress, depression, and CDD in 4 subcounties across rural western Kenya. The study also identified several predictors of CDD related to child, maternal, and household characteristics that shed light on the intricate relationship between maternal mental health and CDD. The findings show that the mean DDS was 4.40. Although our primary analysis was based on the continuous variable of number of food groups consumed, when using the indicator of minimum dietary divers, we found a prevalence of 55% compared with the national average of 37% in Kenya based on the Demographic and Health Survey [35]. Approximately 20.14% of mothers had elevated parenting stress, whereas 41% of mothers were at risk of depression. The adjusted regression analysis revealed that maternal parenting stress is associated with lower CDD, whereas no association was found between maternal depression and CDD. CDD was significantly influenced by various sociodemographic factors. Older children had higher diversity scores, whereas male children had lower scores. Lower dietary diversity was found in children from lower-wealth and food-insecure households. In contrast, higher maternal social support was associated with better dietary diversity outcomes. These findings offer valuable contextual information for social and household contexts of maternal mental well-being and child nutrition.

Most of the available evidence focuses on the associations between maternal mental health and child feeding practices and comes from HICs. This study contributes to the evidence-base using novel measures of parenting stress and CDD and documented evidence from the baseline data from 2 trials in rural western Kenya. Our study underscores the detrimental impact of maternal parenting stress on CDD. The significant negative association between maternal parenting stress and CDD suggests that children of mothers with parenting stress are less likely to meet dietary diversity requirements for the children. Hence, maternal well-being plays a crucial role in shaping CDD and, consequently, children’s nutritional status. This aligns with previous studies that have documented the adverse effects of parental stress on various aspects of feeding behaviors, including child development in HICs such as the United States, United Kingdom, and Australia [8,51,52]. For example, a recent systematic review and meta-analysis showed that parenting stress was associated with suboptimal feeding practices and unresponsive feeding styles among the mothers in HIC settings [8]. This finding underscores the importance of conducting parenting stress screenings to identify mothers experiencing parenting stress when selecting the target population for an intervention.

Our study found no significant association between maternal depression and CDD after adjusting for covariates. This contrasts with some earlier findings suggesting a negative impact of maternal depression on child nutrition in different LMICs, including sub-Saharan Africa [7,17,53,54]. Nevertheless, it is worth noting that the broader body of evidence on this association is mixed. For example, a cross-sectional study in a low-income urban setting in Kenya found that compared with nondepressed mothers, depressed mothers were less likely to practice exclusive breastfeeding and had a higher odds of having an underweight infant [37]. Similarly, studies in rural settings of Ethiopia, Bangladesh, Nepal, and India also found maternal depression to be associated with lack of dietary diversity, inappropriate complementary feeding, and suboptimal breastfeeding [[55], [56], [57], [58]]. On the contrary, studies in Ghana, Demographic Republic Congo, and Nepal found no association between maternal depression and CDD, feeding practices, and complementary feeding [22,59,60]. Our results highlight the need for additional studies to clarify the links between maternal depression and child feeding behaviors, preferably using longitudinal study designs and in rural context.

Our study also highlights the importance of considering several predictors of CDD. As a covariate, we observed a positive association between maternal social support and CDD, indicating that higher levels of maternal social support are linked to greater dietary diversity among children, which aligns with previous studies [52,[61], [62], [63], [64]]. This finding corroborates the importance of seeking or integrating maternal social support or social networks as a means of improving child diet and other health outcomes in child nutrition interventions [65,66]. Our findings confirm that such strategies are critical to adopt, especially in the context of maternal depression and child feeding practices. In addition, socioeconomic gradients have been well-documented for child malnutrition including dietary diversity [[67], [68], [69]]. Consistent with previous studies in Africa [70,71], our study found disparities in CDD based on demographic factors, household wealth, maternal education level, and food insecurity. Specifically, the older children tend to have higher dietary diversity compared with the younger children, which is aligned with an Ethiopian study [72], may be due to the cultural beliefs that discourage introducing cereals, legumes, and animal source foods to the young children [73,74].

Our analysis also shows male children receive less diverse diets than females, contrasting with findings from a study in 3 sub-Saharan African countries including Gambia, Liberia, and Rwanda [75]. However, our finding is aligned with a study that looked at the gender differences in diet in 8 sub-Saharan African countries including Burkina Faso, Ethiopia, Ghana, Nigeria, Tanzania, and Uganda, which found female adolescents were more likely to have diversified foods than male adolescents [76]. Although diet requirements are similar for both male and female children, irrespective to their age groups, our findings reconfirm the importance of increasing parental awareness and promoting equitable feeding behavior [77].

Our findings underscore the important role of maternal education in shaping CDD. Across both models, even modest educational attainment was significantly associated with better dietary diversity, suggesting that improving maternal education may be a key pathway to enhancing child nutrition outcomes. The findings align with studies in Kenya, where maternal education significantly predicted CDD in Kwale County [78]. Across sub-Saharan Africa, similar patterns emerge as higher maternal education correlates with better child feeding practices and reduced malnutrition, consistent with evidence from the Democratic Republic of Congo and multicountry analyses [79].

Moreover, our analysis underscored the detrimental impact of food insecurity and low socioeconomic status on CDD. Households experiencing moderate and high levels of food insecurity exhibited significantly lower dietary diversity among children. In addition, our analysis found that, compared with the households with lowest quintile, children in highest, fourth, and middle quintiles are more likely to feed minimum acceptable diet. These findings align with a regional study that generated evidence from demographic and health surveys from 2010 to 2020 in 33 sub-Saharan Africa, including Kenya [71]. This emphasizes the need for holistic interventions for improving child diet and maternal mental health, by exploring integrated pathways to address psychosocial aspects, alongside with food insecurity, and poverty alleviation [80,81]. Our study provides a data-driven process that can be used to identify mothers at risk of maternal mental health issues, households with food insecurity, and poor child diet, in order to hone recruitment and design of such interventions. These findings highlight the importance of addressing social and economic determinants by focusing on households experiencing food insecurity and lower socioeconomic status.

### Limitations

When interpreting the findings of this study, a few limitations should be considered. First, the indicators of focus in this study—including maternal depression, parenting stress, and CDD—were measured through maternal reports. As a result, these measures may be subject to recall and social desirability bias. Second, to measure CDD, the caregiver was asked about their child’s feeding practices at the food group level where they were asked if they had fed their child any foods from each designated food group in the past 24 h. Although the enumerators provided a list of examples for each of the food groups and had undergone training to ensure the respondent’s adequate understanding, this approach may be subject to additional measurement errors when compared with a 24-h dietary recall if the respondent incorrectly categorized the foods they fed their child in the past day. A key limitation of our food insecurity measurement is that we did not use the full, validated scale; instead, we adapted only 2 items, which may not fully capture food insecurity. Additionally, because baseline assessments were conducted at different times, seasonal variation in food insecurity may have introduced measurement bias. Pooling baseline data from 2 studies conducted at different times may introduce heterogeneity due to differences in participant demographics. To minimize bias, we adjusted for cluster, geographic location, and key demographic variables in our models. Finally, the cross-sectional design of this study prevents the inference of any causal relationships between maternal mental health and CDD.

In conclusion, our findings contribute to the growing body of literature on maternal mental health and child nutrition from a LMIC (Kenya), where the relationship between maternal mental health and child nutrition is understudied. Our study reveals that maternal parenting stress influenced CDD, although maternal social support may serve as a protective factor mitigating the adverse effects of maternal mental health on CDD. The findings highlight the need for a comprehensive and tailored intervention to enhance maternal social support, increase parental awareness of equitable nutritional needs for male and female children, and promote equitable parental responsibilities in household chores to address maternal mental health and improve CDD. The findings also advocate for an equity-based targeting approach to identify vulnerable households and promote gender equity, while promoting dietary diversity for the children. Beyond short-term interventions, our findings also offer insights to help define strategies for sustainable change in the psychosocial factors influencing child diet. Strategies that provide ongoing mental health support, strengthen social networks, alleviate household food insecurity, advance gender equity within households, and ensure fair access to resources have the potential to uplift maternal well-being and promote better nutrition for children.

## Author contributions

The authors’ responsibilities were as follows – MAK, JKM, ZS, AG, JJ: conceived the study; JKM, MO, JJ: curated the data; MAK, ZS, JKM, JJ: performed formal analysis; JKM, MO, JJ: were responsible for funding acquisition; JKM, MO, JJ: performed investigations; MAK, JKM, ZS, AG, MO, JJ: performed methodology; JKM, MO, JJ: administered the project; JJ: was responsible for resources and supervised the study; JJ, MAK: performed software analysis; ZS, JKM, JJ: validated the study; MAK, AG: visualized the study; MAK: wrote the original draft; MAK, JKM, ZS, AG, MO, JJ: reviewed and edited the manuscript; and all authors: have read and approved the final manuscript.

## Data availability statement

All data relevant to the study are included in the article or uploaded as supplementary information. However, the data set can be made available upon reasonable request to the corresponding author.

## Funding

This study received funding from Episcopal Relief and Development and the Conrad N. Hilton Foundation. The views expressed are solely those of the authors and do not reflect the perspectives of the funders. The funders were not involved in the study’s design, data collection, analysis, interpretation, the writing of this manuscript, or selecting journal to publish the results.

## Conflict of interest

JJ, JKM, and MO reports financial support was provided by Episcopal Relief and Development and by Conrad N Hilton Foundation. The other authors declare no competing interests.
