# Supplementary material for: Maternal Mental Health and Child Dietary Diversity in Rural Kenya: Findings From a Pooled Analysis of 2 Baseline Studies
Source: Curr Dev Nutr. 2025 Jun 24;9(7):107497. doi: 10.1016/j.cdnut.2025.107497 (PMC12296480; doi:10.1016/j.cdnut.2025.107497)
Supplement: multimedia component 1 [file mmc1.docx]

**Maternal mental health and child dietary diversity in rural Kenya: Findings from a pooled analysis of two baseline studies**

Md Abul Kalam.

Supplementary file 1: Comparison of socio-demographics, exposures and outcomes by MTM and ChildFund trial

| **Variable ​** | **Mean; SD​; Range; n (%)*** | | | |
| --- | --- | --- | --- | --- |
|  | **Total** | MTM Trial | ChildFund Trial | p-value# |
| **Child Age (in months)** | 13.68; ±5.02; 6-25 | 12.09; ±3.56; 6-19 | 14.97; ±5.63; 6-25 | <0.001 |
| **Child Sex** | | | | |
| *Female* | 353 (51.16) | 172 (55.66) | 181 (47.51) | 0.033 |
| *Male* | 337 (48.84) | 137 (44.34%) | 200 (52.49) |  |
| **Any breastfeeding** | | | | |
| *Yes* | 525 (76.09) | 221 (71.52) | 304 (79.79) | 0.011 |
| *No* | 165 (23.91) | 88 (28.48) | 77 (20.21) |  |
| **Siblings under 5** | | | | |
| *No sibling* | 446 (64.64) | 217 (70.23) | 229 (60.10) | 0.001 |
| *1 sibling* | 226 (32.75) | 90 (29.13) | 136 (35.70) |  |
| *2 siblings* | 18 (2.61) | 2 (0.65) | 16 (4.20) |  |
| **Maternal Age (in years)** | 27.89; ±5.92; 17-49 | 27.51; ±5.85; 18-46 | 28.20; ±5.96; 17-49 | 0.129 |
| *18-24 years* | 220 (31.88)) | 107 (34.63) | 113 (29.66) | 0.515 |
| *25 - 34 years* | 356 (51.59) | 155 (50.16) | 201 (52.76) |  |
| *35-44 years* | 108 (15.65) | 45 (14.56) | 63 (16.54) |  |
| *45+ years* | 6 (0.87) | 2 (0.65) | 4 (1.05) |  |
| **Maternal Marital Status** | | | | |
| *Never Married* | 91 (13.19) | 46 (14.89) | 45 (11.81) | <0.001 |
| *Married* | 300 (43.48) | 71 (22.98) | 229 (60.10) |  |
| *Cohabitating* | 251 (36.38) | 165 (53.40) | 86 (22.57) |  |
| *Other* | 48 (6.96) | 27 (8.74) | 21 (5.52) |  |
| **Maternal Education** | | | | |
| *No education ​* | 5 (0.72) | 3 (0.97) | 2 (0.52) | <0.001 |
| *Some primary school* | 173 (25.07) | 42 (13.59) | 131 (34.38) |  |
| *Primary school completed ​* | 283 (41.01) | 128 (41.42) | 155 (40.68) |  |
| *Secondary school completed ​* | 229 (33.19) | 136 (44.01) | 93 (24.41) |  |
| **Wealth Quintile (1 - 5)** | | | | |
| *Lowest* | 133 (19.28) | 60 (19.42) | 73 (19.16) | 1.000 |
| *Second* | 140 (20.29) | 63 (20.39) | 77 (20.21) |  |
| *Middle* | 138 (20.00) | 62 (20.06) | 76 (19.95) |  |
| *Fourth* | 142 (20.58) | 63 (20.39) | 79 (20.73) |  |
| *Highest* | 137 (19.86) | 61 (19.74) | 76 (19.95) |  |
| **Food Insecurity** | | | | |
| *None* | 367 (53.19) | 212 (68.61) | 155 (40.68) | <0.001 |
| *Low* | 110 (15.94) | 26 (8.41) | 84 (22.05) |  |
| *Moderate* | 106 (15.36) | 39 (12.62) | 67 (17.59) |  |
| *High* | 107 (15.51) | 32 (10.36) | 75 (19.69) |  |
| **Child dietary diversity (0-8)** | 4.40; ±1.63 ; 1-8 | 4.41; ± 1.63; 1-8 | 4.38; ± 1.71; 1-8 | 0.788 |
| **Child minimum dietary diversity** | |  |  |  |
| *5 and more* | 377 (54.64) | 164 (53.07) | 213 (55.91) | 0.485 |
| *Less than 5* | 313 (45.36) | 145 (46.93) | 168 (44.09) |  |
| **Maternal stress** | | | | |
| *Low Stress (psi <43)* | 551 (79.86) | 291 (94.17) | 260 (68.24) | <0.001 |
| *High Stress (psi >=44)* | 139 (20.14) | 18 (5.83) | 121 (31.76) |  |
| **Depression** | | | | |
| *Low symptoms (cesd<10)* | 410 (59.42) | 200 (64.72) | 210 (55.12) | 0.011 |
| *High symptoms (cesd >= 10)* | 280 (40.58) | 109 (35.28) | 171 (44.88) |  |
| **Fathers' Involvement in household chores** | 1.62; 2.09; 0-7 | 2.01; 2.19. 0-7 | 1.38; 1.99; 0-7 | 0.002 |
| **Social Support** | 3.46; ±0.64; 1-4.92 | 3.35; ±0.62; 1.33-4.92 | 3.54, ±0.65; 1-4.83 | 0.001 |

Abbreviations: SD, Standard Deviation; y, years; cesd, center for epidemiologic studies depression scale; psi, parenting stress index. * Mean, SD, and Range for the continuous variables and % for categorical variables. # for categorical variable p-value was calculated by chi-square tests and for continuous variables, t-tests were used.
